# Sensors for Fluid Leak Detection

**DOI:** 10.3390/s150203830

**Published:** 2015-02-05

**Authors:** Gonzalo Pajares Martinsanz

**Affiliations:** Department of Software Engineering and Artificial Intelligence, Faculty of Informatics, University Complutense of Madrid, 28040 Madrid, Spain; E-Mail: pajares@ucm.es; Tel.: +34-1-394-7546; Fax: +34-1-394-7547

Fluid leak detection represents a problem that has attracted the interest of researchers, but not exclusively because in industries and services leaks are frequently common. Indeed, in water or gas supplies, chemical or thermal plants, sea-lines or cooling/heating systems leakage rates can cause important economic losses and sometimes, what it is more relevant, environmental pollution with human, animal or plant lives at risk. This last issue has led to increased national and international regulations with different degrees of severity regarding environmental conservation.

Early fluid detection represents an important challenge to avoid the problems mentioned above. This special issue was proposed with the aim of attracting new technological developments and methods in sensors based on physical, chemical or biological principles. The following is a summary of works published in this special issue representing the achievements obtained. They are organized according to the main topics and applications addressed in the special issue, which can serve to the reader as a first introduction guide to each work:

1. *CO_2_ and CH_4_ leakage in natural reservoir with micro-seismicity*: In [1] an array of eight short-period borehole geophones, three pressure-temperature sensors and two fluid-sample sensors were deployed with the aim of detecting induced micro-seismicity associated with CO_2_ activity, including injection, within a natural reservoir in the Pembina oil field in Alberta/Canada. The primary objective was to investigate potentially occurring CO_2_-induced seismic signatures on a two-week period framing a substantial CO_2_ and CH_4_ leakage.
2. *Thermal power plants*: An autonomous robotic system is designed in [2] to perform pipeline inspection for early detection and prevention of leakages in solar thermal power plants. A thermographic camera provides the required information to detect leakages in collectors and pipelines and also for tracking purposes. Experiments were conducted in solar plants at the Torresol Energy Investments S.A. facilities in San José del Valle, Spain. The solar field is composed of nearly 7500 parabolic cylinder collectors that transport a Heat Transfer Fluid (HFT), which absorbs the solar energy. HFT circulates at high temperature (∼390 °C) inside the absorber tubes. HTF leakages detection is the final goal of the proposed system.
3. *Water distribution systems*: Sensors, technologies, methods or procedures can be developed to reduce the amount of water leaked in distribution systems. An optimum sensor deployment, based on genetic algorithms, is proposed in [3] for leak detection, isolation and location in water distribution networks. The proposed approach was tested on the water network of Hanoi, Vietnam with 31 demand nodes, one reservoir node and 34 pipes. In [4] a Ground Penetrating Radar is the technology used for leaks detection in water distribution systems, the instrument is equipped with a monostatic antenna operating at a central frequency of 1.5 GHz. The data collected as images are conveniently processed and analyzed based on the identification of vertical and horizontal profiles in the images. Wireless Sensor Networks and Radio Frequency IDentification (RFID) is the technology used in [5] for the design and simulation of a water pipeline leakage monitoring system. The design is based on deploying a group of mobile wireless sensor nodes and allowing them to work cooperatively according to a prescheduled order. Only a node is active at a time while the remainders are sleeping, which are activated based on three kinds of events: location-based, time-based and interrupt-based. Each node, equipped with a pressure sensor, a microcontroller and a RFID reader, records pressures and its location based on its exposure to signals of active RFID tags placed outside of the pipeline surface. The mobile sensor nodes move with the water current from the pipeline source down to the sink where the node is collected and its memory content transferred to a computer for numerical analysis. In the context of water distribution systems at home, numerical models are tested in [6] to determine when an event occurs (tap open, high/low water consumption, seepage). This includes leak detection. The hierarchical hidden Markov model (HHMM) is the method used for the recognition of such events. RFIDs integrated with pressure sensors are embedded in the pipe infrastructure. They collect pressure information and send it along with their IDs to the reader/writer destination node to determine the pattern of the event. The network infrastructure, together with the communication system, configures a water smart home system that allows monitoring the water distribution system and indirectly the leak detection.
4. *Thinning of pipe walls*: Leaks can be caused by degradation in the pipelines' walls, sometimes expressed as a thinning. In this regard, in [7] the analysis of transient fluid pressure signals has been studied and analyzed for detecting thinning in pipelines. This is carried out by placing high speed pressure sensors in contact with the fluid. Analysis based on numerical models is applied to detect the transient flow variations inside the pipeline where the geometry changes because of the thinning.
5. *Buried plastic pipes*: Experimental studies have been conducted in [8] in order to validate leak detection methods in buried plastic pipes using measurements of acoustic pressures (captured with hydrophones), velocities (using geophones), and accelerations (obtained with accelerometers). The leaks generate broadband noise, which propagates along the pipe, both in the fluid and along the pipe-wall either side of the leak, to sensors conveniently located at access points. Because in water plastic pipes the pipe-wall and water exhibit strong acoustic coupling measurements of leak noises can be made in the pipe for leak detection. The differences in the arrival times of the noise at the sensors' locations (time delays) determine the position of the leak.
6. *Sewer pipelines*: Closed Circuit Television (CCTV) is the technology used in [9] for detecting leaks in vitrified clay sewer pipes, where the failure level and the position are to be determined. Computer vision techniques based on edge detection and morphology image segmentation operations are used for such purpose. The CCTV was mounted on a robot connected on the ground with a power cable and the Taichung City in Taiwan inspected with the robot traveling between manholes.
7. *Ultrasonic in binary gas*: In [10] an ultrasonic instrument is used to measurement of leaks of a high molecular weight gas (octafluoropropane coolant, C_3_F_8_) into a system that is nominally composed of a single gas (nitrogen) during a long duration (18 month). The sensitivity of the instrument is due to the difference in molecular masses of the two gases in the mixture. The impact of variables such as temperature and pressure on the accuracy of the measurement is analyzed. Ultrasonic bursts are propagated in a sealed tube designed to provide a smooth flowing gas region between two transducers. Each transducer is comprised of a thin, Au plated foil stretched over a spirally grooved conductive disk. The foil is held at ground potential and the disk is biased between 100 Vdc and 360 Vdc. A 50 kHz pulse train modulates the transducer bias voltage, exciting the diaphragm to transmit an acoustic wave in response to the fluctuating electric field. A micro-controller creates gated pulses and measures the time of fight pulses between the transmitter and receiver transducers.
8. *Offshore Pipelines*: Girth-welds in offshore submerged pipelines has been numerically analyzed in [11] because their potential to cause leaks. Eight piezoelectric transducers (sensors/actuators), enclosed by a special waterproof coating, were bonded to an aluminum pipe in either sides of the girth-welds. The electrical connections were sealed manually with silicon and secured by heat shrink sleeves. Two excitation methods were applied for conducting the damage detection trials during the experiments: (a) impact, with a waterproofed pneumatic hammer; (b) chirp waves ranging in 10–5000 Hz, produced by using one of the piezoelectric transducers as actuator and conveniently amplified.
9. *Pressure sensors*: Pressure changes in fluid distribution networks may be evidences of malfunctioning and perhaps of leaks. A clamped-clamped beam-type piezoelectric vacuum pressure sensing device is designed in [12]. The piezoelectric element together with a self-actuating and a self-sensing microresonator detect the damping ratio of the gas, which allows enabling the calculation of the pressure of the vacuum system. The sensing element comprises a piezoelectric ceramic lead zirconate titanate (PZT) layer, a substrate and two pairs of electrodes. A pair is used to apply a sinusoidal voltage signal and the second pair receives the vibrations. The received vibrations are finally converted to electric energy using the positive piezoelectric effect. The output voltages, which varied under different gases viscosity and vacuum pressures, are measured by the device.

## References

[1] Martínez-Garzón P., Bohnhoff M., Kwiatek G., Zambrano-Narváez G., Chalaturnyk R. (2013). Microseismic Monitoring of CO2 Injection at the Penn West Enhanced Oil Recovery Pilot Project, Canada: Implications for Detection of Wellbore Leakage. Sensors.

[2] Ibarguren A., Molina J., Susperregi L., Maurtua I. (2013). Thermal Tracking in Mobile Robots for Leak Inspection Activities. Sensors.

[3] Casillas M.V., Puig V., Garza-Castañón L.E., Rosich A. (2013). Optimal Sensor Placement for Leak Location in Water Distribution Networks Using Genetic Algorithms. Sensors.

[4] Ayala-Cabrera D., Herrera M., Izquierdo J., Ocaña-Levario S.J., Pérez-García R. (2013). GPR-Based Water Leak Models in Water Distribution Systems. Sensors.

[5] Almazyad A.S., Seddiq Y.M., Alotaibi A.M., Al-Nasheri A.Y., BenSaleh M.S., Obeid A.M., Qasim S.M. (2014). A Proposed Scalable Design and Simulation of Wireless Sensor Network-Based Long-Distance Water Pipeline Leakage Monitoring System. Sensors.

[6] Nasir A., Hussain S.I., Soong B.-H., Qaraqe K. (2014). Energy Efficient Cooperation in Underlay RFID Cognitive Networks for a Water Smart Home. Sensors.

[7] Tuck J., Lee P. (2013). Inverse Transient Analysis for Classification of Wall Thickness Variations in Pipelines. Sensors.

[8] Almeida F., Brennan M., Joseph P., Whitfield S., Dray S., Paschoalini A. (2014). On the Acoustic Filtering of the Pipe and Sensor in a Buried Plastic Water Pipe and Its Effect on Leak Detection: An Experimental Investigation. Sensors.

[9] Su T.-C., Yang M.-D. (2014). Application of Morphological Segmentation to Leaking Defect Detection in Sewer Pipelines. Sensors.

[10] Bates R., Battistin M., Berry S., Bitadze A., Bonneau P., Bousson N., Boyd G., Bozza G., Crespo-Lopez O., Riva E.D. (2014). Implementation of Ultrasonic Sensing for High Resolution Measurement of Binary Gas Mixture Fractions. Sensors.

[11] Razi P., Taheri F. (2014). A Vibration-Based Strategy for Health Monitoring of Offshore Pipelines' Girth-Welds. Sensors.

[12] Wang B.-Y., Hsieh F.-C., Lin C.-Y., Chen S.-E., Chen F.-Z., Wu C.-C. (2014). Development of a Piezoelectric Vacuum Sensing Component for a Wide Pressure Range. Sensors.

